# Strain imaging in abdominal aortic aneurysms using bistatic dual-aperture ultrasound

**DOI:** 10.1038/s41598-025-23710-8

**Published:** 2025-11-13

**Authors:** Vera H. J. van Hal, Lisanne T. F. Passier, Marc R. H. M. van Sambeek, Hans-Martin Schwab, Richard G. P. Lopata

**Affiliations:** 1 Photoacoustics & Ultrasound Laboratory Eindhoven (PULS/e), Eindhoven University of Technology, P.O. Box 513, 5600 MB Eindhoven, The Netherlands; 2 Department of Vascular Surgery, P.O. Box 1350, 5602 ZA Eindhoven, The Netherlands

**Keywords:** Abdominal aortic aneurysm, Ultrasound, Multi-aperture, Strain imaging, Biomedical engineering, Aneurysm, Imaging techniques

## Abstract

Knowledge of the full geometry of abdominal aortic aneurysms (AAA) and local, mechanical wall parameters using ultrasound (US) can contribute to a better assessment of the AAA’s mechanical state, prediction of growth and possible risk of rupture. Such an assessment is currently limited by the anisotropic lumen-wall contrast and the resolution of conventional US. The recent introduction of ultrafast dual-aperture imaging enhances image quality, using two transducers that alternately transmit and receive simultaneously (“bistatic” US). In this study, dual-aperture, bistatic US imaging is assessed in 43 AAA patients. Results were compared to single-aperture ultrafast imaging. Bistatic imaging was demonstrated successfully in 40 patients. Compared to single-aperture imaging, the median wall-lumen generalized contrast-to-noise ratio (gCNR) was significantly increased by 0.13 (+27%). By compounding axial displacements from multiple directions, we show the feasibility of local strain quantification: not only in the vessel wall but also in low contrast regions, such as the intraluminal thrombus (ILT). Multi-aperture ultrasound imaging can provide the clinician with high quality imaging, which contributes towards a better understanding of AAA development and patient-specific rupture risk analysis, by allowing the accurate assessment of tissue properties and function.

## Introduction

An abdominal aortic aneurysm (AAA) is an irreversible local dilation of the abdominal aorta with a prevalence of 1.9% to 18.5% in men and 0% to 4.2% in women, mainly affecting the elderly population (65+)^1^. AAAs are typically asymptomatic until a life-threatening rupture occurs, which is fatal in 75-90% of the cases^2^. Elective endovascular (EVAR) or open surgical repair is performed if the risk of rupture exceeds the risk of surgical intervention. Currently, the diameter is used as a criterion to assess rupture risk. Surgery is considered when the measured aortic diameter exceeds 5.0 cm in women or 5.5 cm in men^3^. This threshold is based on large population studies. However, 2% - 11% of all AAAs rupture before the threshold for surgical intervention is reached^4^. At the same time, large aneurysms that are currently treated according to these guidelines could remain stable over time^5^. This clearly suggests that there are other factors that contribute to the rupture risk other than geometrical ones.

From a biomechanical perspective, aneurysm rupture happens if the mechanical stresses on the aneurysm wall exceed the local tensile strength of the tissue. Therefore, knowledge of the local mechanical properties of the aneurysm wall can contribute to a better prediction of the rupture risk. On top of that, wall strength and wall stress are affected by the intraluminal thrombus (ILT), which is present in 75% of the patients^6^. The effect of this ILT on AAA development and rupture risk is still controversial. Literature suggests that the presence of ILT reduces AAA wall stress, possibly preventing rupture^7–9^. Other studies show that ILT presence leads to aortic wall weakening^10^. In most previous studies on mechanical analysis of the aneurysm wall, patient specific geometries were made using pre-operative computed tomography (CT). However, it involves the use of ionizing radiation and does not provide temporal information, which is essential for patient-specific characterization of the mechanical properties of the AAA. Alternatively, previous studies indicated that ultrasound (US) imaging, which is already used clinically for the aforementioned diameter measurements, can also be utilized for mechanical analysis, resulting in estimates of wall stress and patient-specific material parameters^11,12^. US speckle-tracking and strain imaging can be used for model personalization^13–15^, from which the wall stiffness can be derived^16^.

Moreover, the local wall deformation, i.e., strain, is an important parameter in itself. Studies have shown that high peak strains may indicate local weakening of the AAA wall^15^. Previous research on AAA strain imaging using US revealed heterogeneous strain patterns across the circumference of the AAA wall^15,17^. In Zottola et al.^18^, the pressure normalized principal strain in AAAs was measured using conventional 2-D US, which showed that these values were independent of AAA size. Recently, Lorenzen et al.^19^ analyzed the local strain patterns measured using conventional 2-D US in fifty patients with an AAA. In general, the highest strains were measured in the anterior wall and on both sides adjacent to the spine. It was found that these patterns were reproducible and that strain pattern clusters may reflect important differences in AAA wall characteristics.

However, the use of conventional US has its limitations as we can only visualize the anterior and posterior side of the AAA wall properly. At the lateral sides, there is a lack of image features and resolution for accurate reconstruction of the geometry and estimation of the wall motion and strain. This is also illustrated in Matthews et al.^20^, where the reproducibility of measurements of aortic diameter in the transverse direction is shown to be significantly lower compared to the anterior-posterior direction.

To gain better insight into the development of AAAs and clinical relevance of these biomechanical parameters, it is necessary to visualize the entire geometry of the aneurysm and quantify wall motion dynamics accurately. To be able to perform more reliable biomechanical measurements, the image quality of the aortic US scans will have to improve significantly. Fundamentally, a larger transducer that scans the aneurysm from multiple directions would produce images with improved contrast and resolution at the lateral sides^21^. To assess the potential of such a system, we recently introduced a dual-aperture imaging setup, based on two curved array transducers, which receive simultaneously upon each transmit event^22^. This technique is called “bistatic” US imaging, a term that originates from radar technology^23^. We have demonstrated its feasibility and performance for both improved image quality and strain estimation in a study with *ex vivo* porcine aortas that were placed in a mock-circulation set-up^22,24^. Next, bistatic imaging was tested in healthy volunteers^25^. This study showed a vast improvement in image quality and revealed more homogeneous strain estimates using multi-aperture US compared to conventional single-aperture US, despite *in vivo* challenges including the low transmit frequency used in abdominal imaging, and the occurrence of aberration and clutter^25^.

In this study, dual-aperture, high-frame rate, US imaging and strain imaging are demonstrated and evaluated in 43 patients with an AAA. The US image quality obtained with the proposed approach is compared to that obtained with conventional imaging with a single transducer. Next, motion tracking of the abdominal aortic wall is performed to estimate local circumferential strains, which is the principal direction of deformation of the arterial wall. With dual-aperture imaging, displacement estimations from multiple directions can be combined, taking advantage of the phase information in the propagation direction of each transducer. To this end, the methods employed for displacement compounding^22,25^, were adapted to take into account the local geometry of AAAs, as local radial and circumferential directions will differ substantially for each patient. In patients with an ILT, the feasibility of local strain quantification was not only assessed inside the vessel wall, but also in the thrombus itself. Local strain quantification within the ILT is more challenging compared to strain estimation within the AAA wall, due to the limited echogenicity of the ILT. This is why local strain analysis inside the ILT has not been performed in literature before. Finally, the strain estimates were compared against conventional US imaging in terms of precision, and the distribution of local strain values across the circumference of the AAA wall using dual-aperture US imaging was analyzed.

**Fig. 1:**
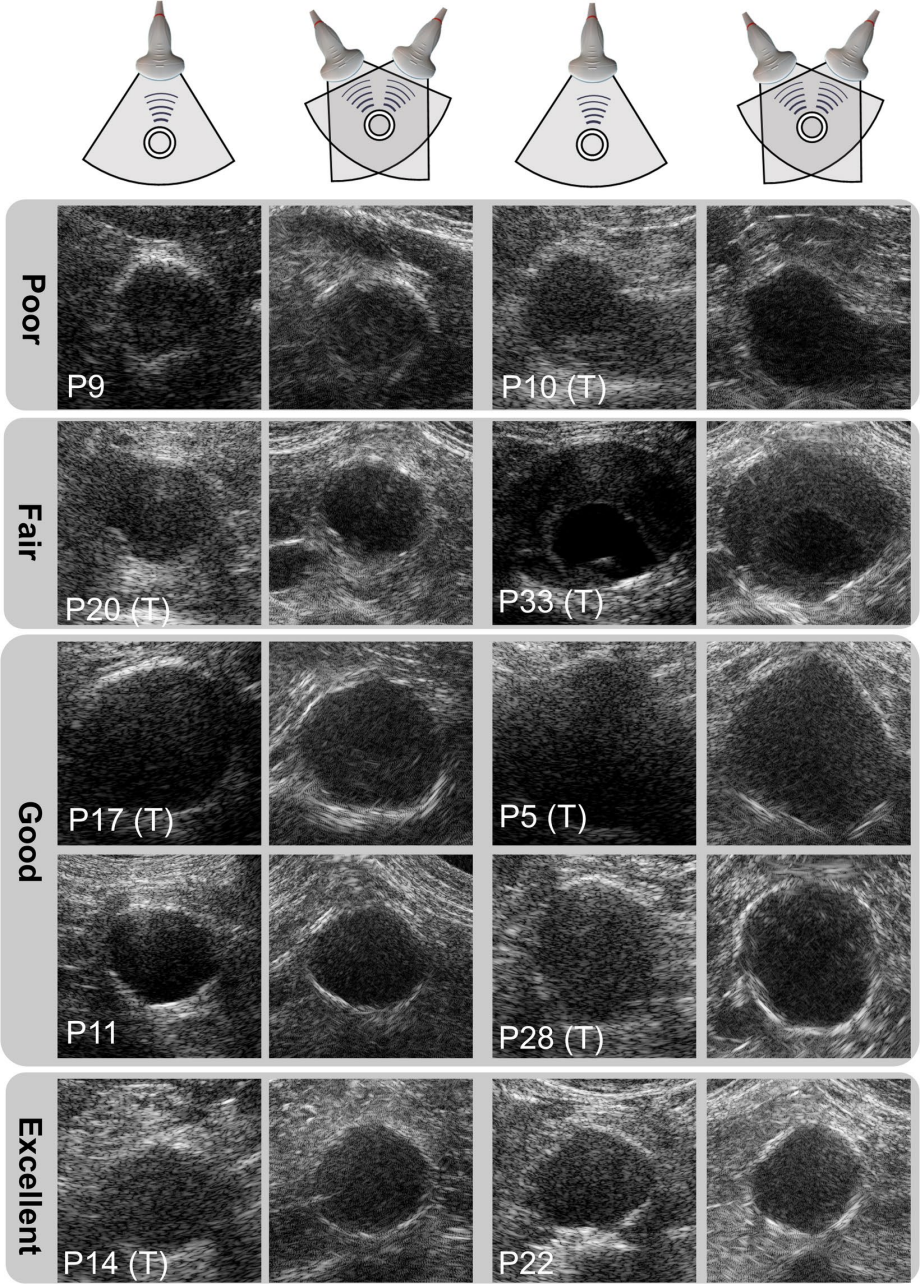
Zoomed-in ultrasound images of the abdominal aortic aneurysm using single-aperture ultrasound imaging (left) and bistatic coherent dual-aperture ultrasound imaging (right). All images have the same size of 7$\times$7 cm. The (T) behind the patient number indicates whether ILT is present. Images are displayed according to the average image quality score of the bistatic US images, as scored by five professionals. The dynamic range of all images is 55dB. Contrast stretching is applied with $\alpha$=1.3.

## Results

In 40 out of the 43 patients, the acquisitions were performed successfully. The datasets of three patients were excluded because the visibility of the AAA on the US image was obstructed by the presence of bowel gas. The baseline patient and aneurysm characteristics are shown in Table 1.

The study included 37 men and 3 women. The mean diameter of the AAA was 44.8 mm (sd: 8.1 mm). ILT was observed in 25 patients (62.5%), with a total ILT thickness ranging from 11 to 33 mm. In 7 patients (17.5%), no ILT was observed. For 8 patients (20.0%), it was unclear whether there was any ILT, as no Color Doppler US scan was available.

**Table 1:** Baseline characteristics.

Patient characteristics	
Patients with AAA	N=40
Gender	
Male	37 (92.5%)
Female	3 (7.5%)
Age, years (mean)	75.2 (sd: 7.1)
Weight, kg (mean)	79.5 (sd: 10.6)
Length, cm (mean)	175.5 (sd 6.9)
BMI, $kg/cm^2$	25.8 (sd: 3.0)
Aorta characteristics	
AAA diameter, mm (mean)	44.8 (sd: 8.1)
Thrombus in AAA	
Patients with ILT	25 (62.5%)
Lumen diameter, mm (range)	19-40
Total ILT thickness, mm (range)	11-33
Patients without ILT	7 (17.5%)
Not sure about presence of ILT	8 (20.0%)

### Image quality

A visual comparison between the single-aperture and the bistatic dual-aperture acquisitions is presented in Fig. 1. The images are displayed according to the average image quality score (poor, fair, good or excellent) of the bistatic US images, as scored by five professionals. Using bistatic dual-aperture US imaging, a larger part of the vessel wall circumference can be visualized through specular reflections. Signifcant image improvement in the visibility and appearance of the surrounding structures, such as the vena cava, abdominal muscles, and spine can be observed when combining the four signals in bistatic imaging (see Figs. 1 and 7). Note that the speckles in the bistatic US image are oriented in different directions, resulting from the combination of the individual US signals. Hence, the point scatterers have a more round or cross-shaped appearance, compared to conventional US imaging where the point scatterers have an ellipsoid shape.

In Fig. 1, the (T) behind the patient number indicates whether ILT is present, which is determined from Color Doppler US imaging or CT data from the same patient (if available). The visibility of the ILT varies substantially between patients and between single-aperture US and bistatic dual-aperture US acquisitions. In some patients, a slight increase in ILT visibility could be observed, for instance in P17. However, in most cases, the increase in vessel wall visibility due to specular reflections was more pronounced than the improvement in ILT visibility. Other examples where the ILT is well visible on the US images using both acquisitions are P20 and P33. In P10, there seems to be a decrease in ILT visibility using bistatic dual-aperture US imaging, compared to single-aperture US. Thus, the lumen-thrombus contrast remains limited and the ILT may also not be visible at all, such as in P5 and P14, even with bistatic coherent dual-transducer US imaging.

**Fig. 2:**
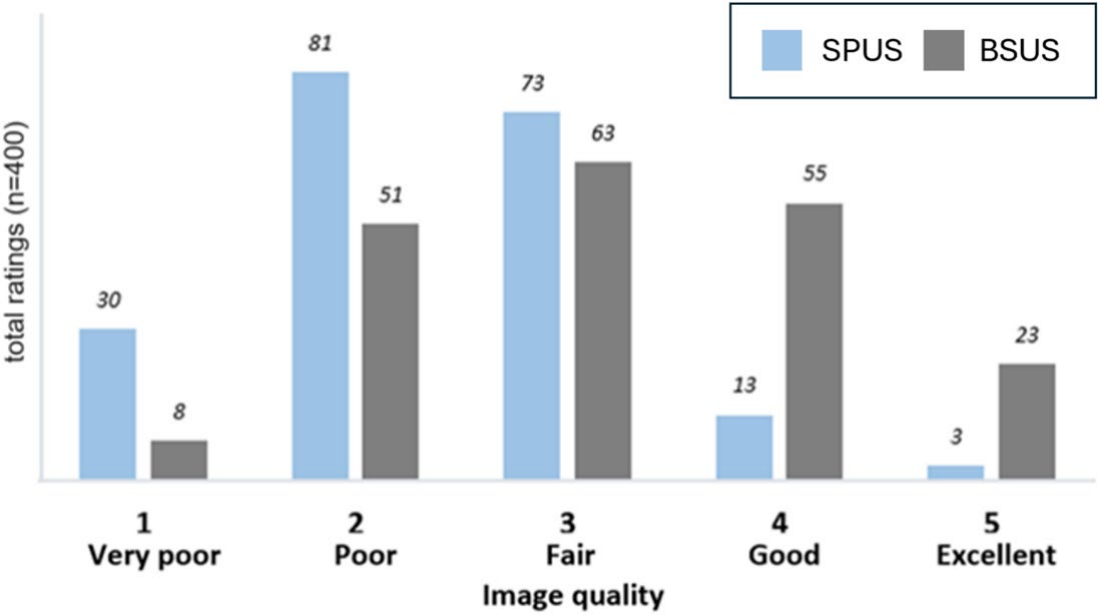
Comparison of the qualitative image quality between conventional single-probe (SPUS) and bistatic ultrasound acquisitions (BSUS).

Five professionals scored 40 single-aperture US and 40 bistatic US images for image quality, resulting in 200 single-aperture US and 200 bistatic US ratings. The median image quality score was 2 (IQR: 1) using single-aperture US and 3 (IQR: 2) for bistatic US, with a significant difference between the scores (p<0.001). The frequency of all the scores is presented in Fig. 2. A total of 111 ratings (55.5%) of single-aperture US images were scored as very poor or poor image quality while 89 ratings (44.5%) were rated as fair, good, or excellent in image quality. Bistatic US showed clear improvement, as 59 images (29.5%) were scored as very poor or poor image quality and 141 images (70.5%) were rated as fair, good or excellent in image quality.

**Fig. 3:**
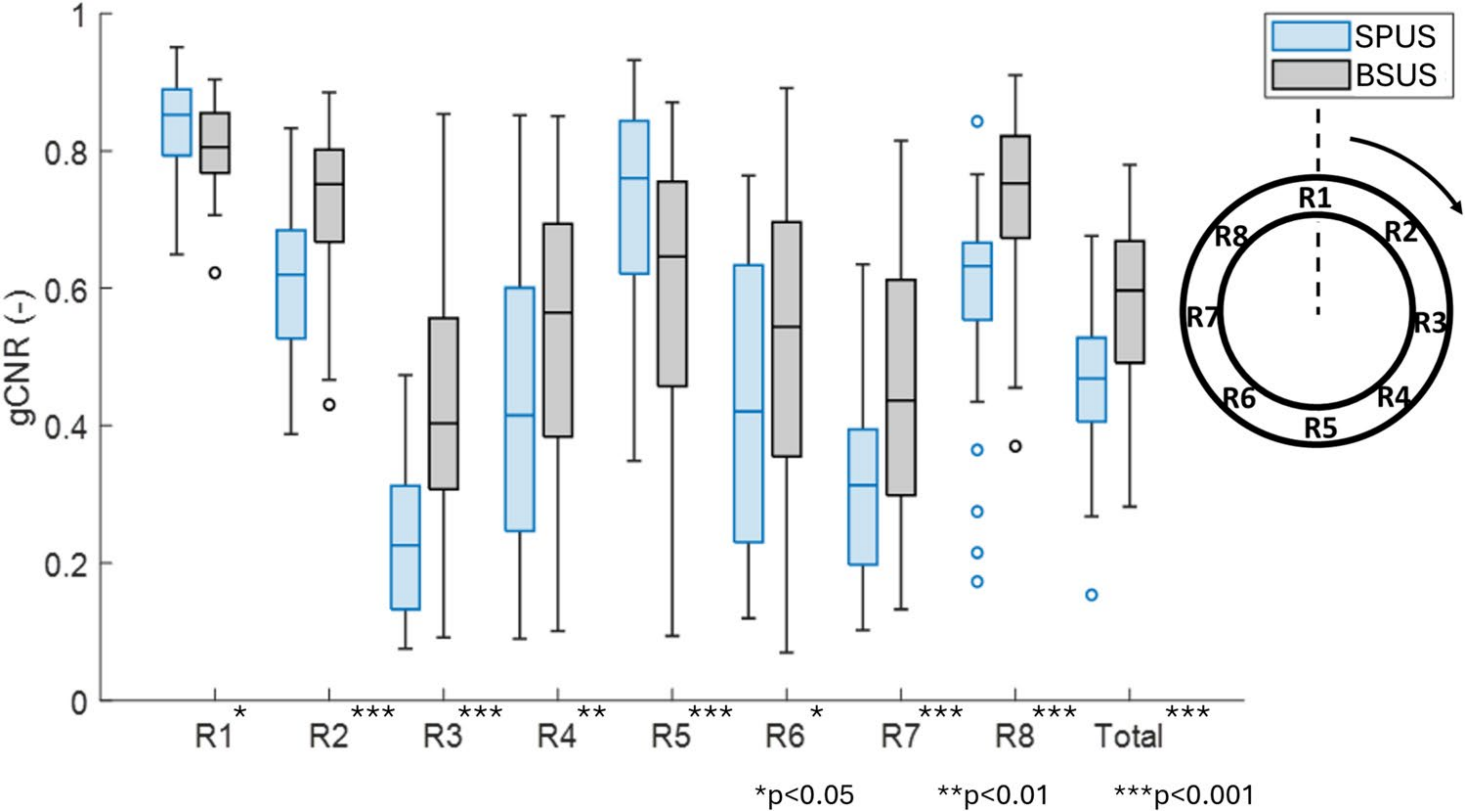
Regional analysis of the generalized contrast-to-noise ratio (gCNR) between the vessel wall and the lumen. Single-probe (SPUS) and bistatic dual-probe ultrasound (BSUS) acquisitions are compared. Significant differences are indicated with an asterisk in the label.

The improvement in AAA visibility was also reflected in the generalized contrast-to-noise ratio (gCNR) measurements. The results of gCNR measurements in all patients (N=40) are quantified and shown in Fig. 3. When considering the total circumference of the aortic wall ($R_{total}$), a significant median increase of 0.13 (+27%) was observed when using bistatic dual-aperture US compared to single-aperture US (p<0.001). The average gCNR using bistatic ultrasound imaging was equal to 0.58 ± 0.12 and the intra-variability range was equal to 0.56 ± 0.12 using the segmentations with the lowest gCNR and 0.59 ± 0.12 using segmentations with the highest gCNR. For single-aperture ultrasound imaging, the average gCNR was equal to 0.47 ± 0.10 with an intra-variability range of 0.46 ± 0.10 using segmentations with the lowest gCNR and 0.48 ± 0.10 using segmentations with the highest gCNR. When analyzing the regions individually, the gCNR is significantly higher using bistatic dual-aperture US imaging (p$\le$0.013) in regions R2 to R4, and R6 to R8. In regions R1 and R5, the gCNR is significantly higher using single-aperture US (p$\le$0.016).

### Strain imaging

**Fig. 4:**
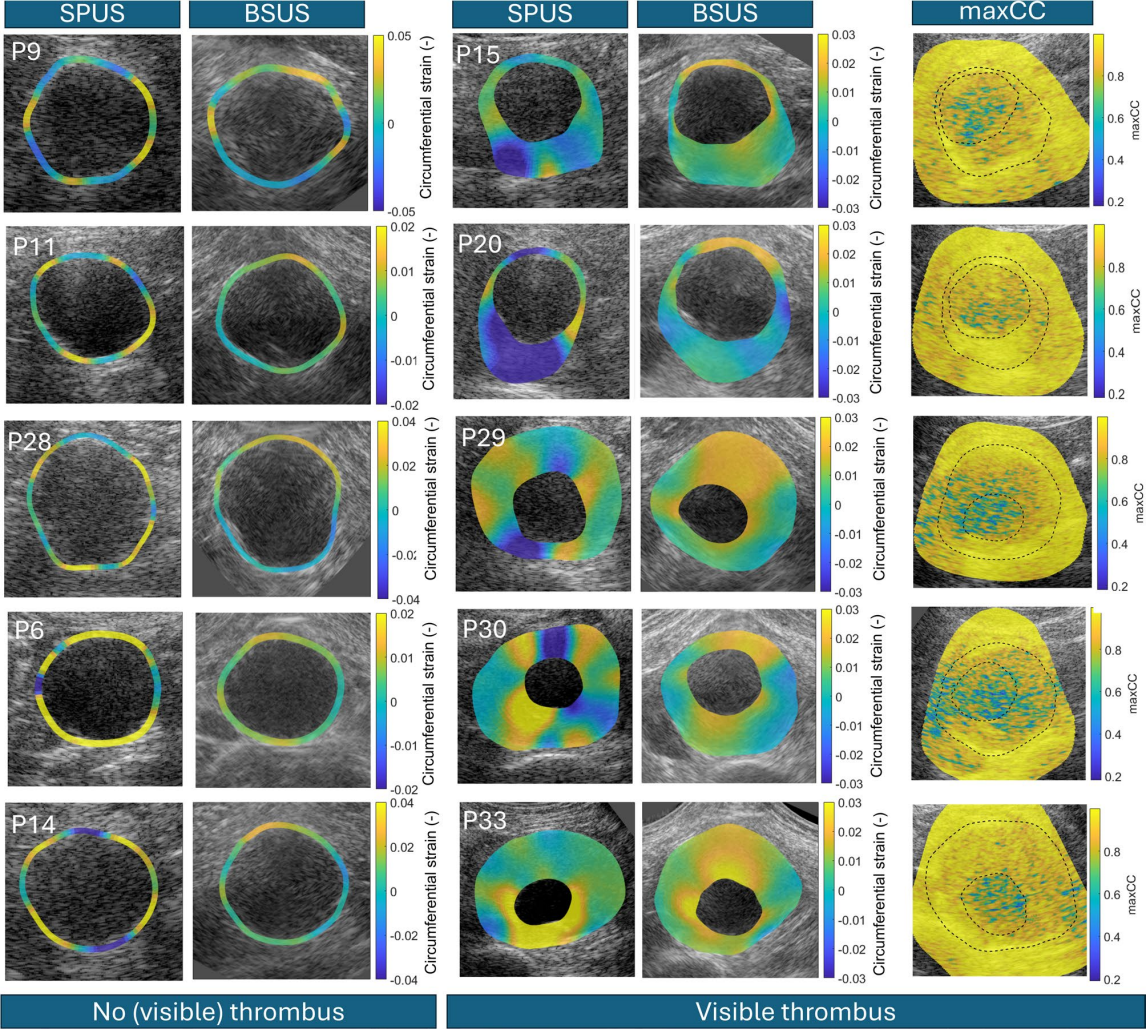
Circumferential strain results at end-systole using single-aperture ultrasound imaging (SPUS) and bistatic dual-aperture ultrasound imaging (BSUS). Left column: results for aneurysms without a visible thrombus. Images are displayed with increasing image quality score from top to bottom. Right column: strain results for aneurysms with visible thrombus. All bistatic US images in this column were scored with fair image quality. Far right: maximum cross-correlation coefficients (maxCC) of the block-matching during systole inside the vessel wall and thrombus in the single-transducer configuration of the bistatic acquisition. The dashed lines indicate the inner and outer border of the segmentation. The images were cropped to fit the aneurysm size: 5$\times$5 cm (P9), 6$\times$6 cm (P6, P11, P14, P15, P20), 7$\times$7 cm (P28, P29, P30), 8$\times$8 cm (P33). A video of the measured circumferential strain using BSUS in P15 and P33 is available as supplementary material.

In 27 out of 40 patients, motion of the aneurysm wall was followed over time. In the other 13 patients, successful motion tracking was performed not only in the aneurysm wall, but also inside the ILT. An overview of the strain results using single-aperture US imaging and bistatic dual-aperture US imaging is shown in Fig. 4.

**Fig. 5:**
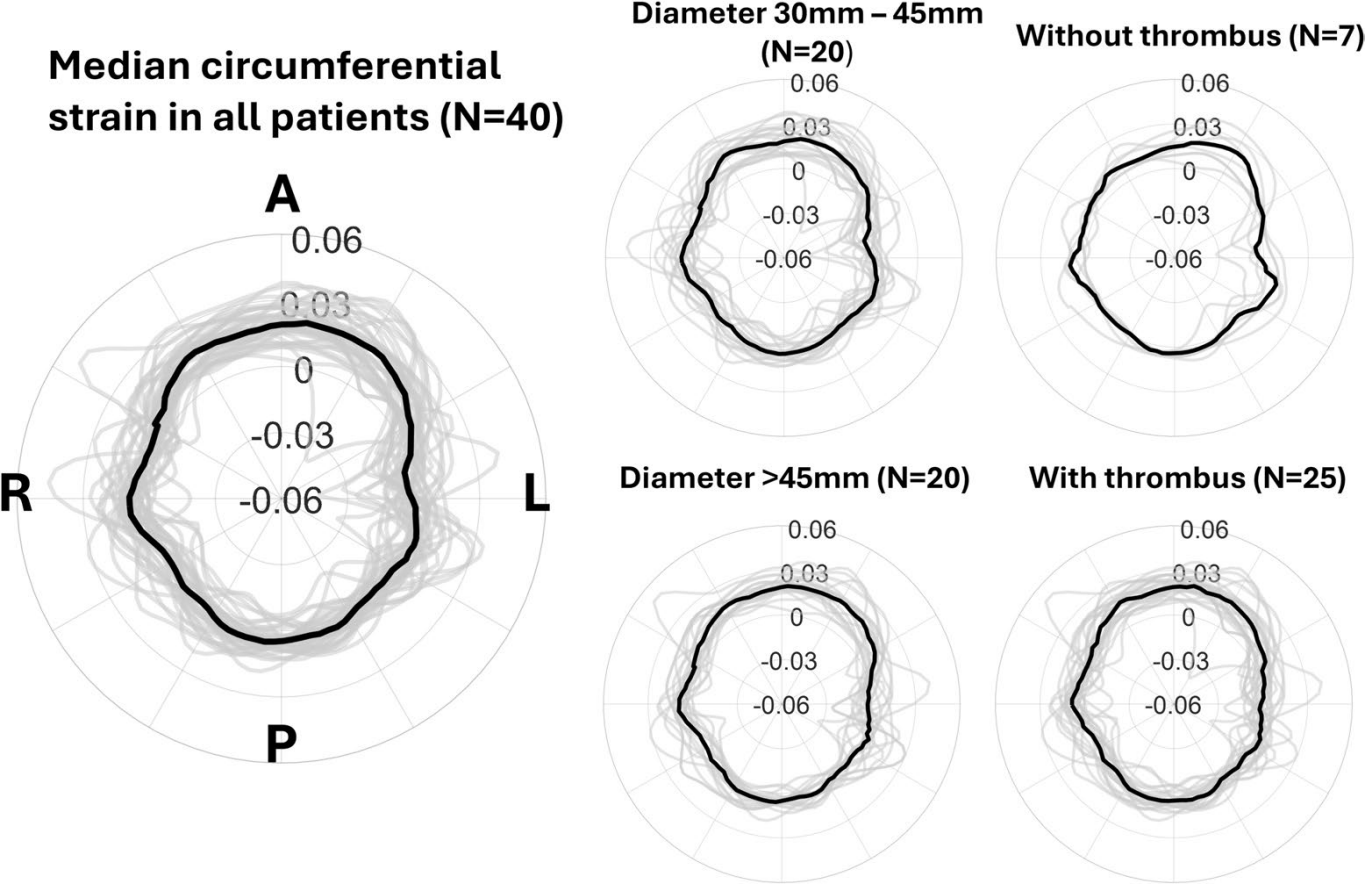
Polar plots illustrating aligned median circumferential strain patterns in the aneurysm wall in all patients. The gray lines show the individual traces. The letters A, L, P, R indicate the anterior, patient left, posterior and patient right sides, respectively.

The resulting strain patterns using single-aperture US imaging display large heterogeneities and regions of very high or very low circumferential strain, which can be explained by lateral motion drift. In bistatic dual-aperture US imaging, only the axial displacements were used during displacement compounding, while the lateral displacements were not taken into account. Therefore, the strain patterns using bistatic US imaging were distinctly different compared to single-aperture US, displaying more homogeneous strain results. Local heterogeneities are still present, however, due to the decrease in motion drift, this can reflect the underlying tissue properties.

The use of regularization in single-aperture US strain imaging significantly reduced the mean motion drift across the entire circumference of the aorta from 0.72 mm ± 0.23 mm to 0.36 mm ± 0.18 mm (p<2·10^-16^). Using bistatic dual-aperture US imaging, the total motion drift was further decreased significantly to 0.14 mm ± 0.19 mm without regularization (p = 9·10^-15^) and to 0.11 mm ± 0.11 mm with regularization (p = 7·10^-9^), clearly demonstrating the substantial benefit of using bistatic dual-aperture US imaging to estimate local motion dynamics in the AAA wall. The decrease in motion drift was also reflected by a significant increase in SNRe^circ^ of 9.5 dB over all 40 patients (p = 1.4·10^-6^), from -17.3 dB ± 10.8 dB using single-aperture US to -7.9 dB ± 7.3 dB using bistatic dual-aperture US. With the use of regularization in both single-aperture US and bistatic dual-aperture US strain imaging, this increase was equal to 7.6 dB (p = 9.2·10^-6^), from -11.4 dB ± 9.9 dB using single-aperture US to -3.8 dB ± 6.7 dB using bistatic dual-aperture US. The resulting SNRe^circ^ was larger using bistatic dual-aperture US imaging without the use of regularization, compared to what could be achieved with regularization in single-aperture US.

In 13 out of 25 patients with ILT, successful strain imaging could be performed in the entire AAA. In the right column of Fig. 4, the maximum cross-correlation coefficient in the single-transducer configuration of the bistatic acquisition is plotted during systole, for several patients in which motion tracking was performed in the vessel wall and ILT. There was a clear difference in maximum cross-correlation between regions in the ILT (values of around 0.8) and the lumen (around 0.4), demonstrating the feasibility of tracking the ILT over time. However, the maximum cross-correlation values are lower compared to those inside the vessel wall. Especially the regions in the vessel wall with strong specular reflections show high cross-correlation values close to 1, which can be found on the outer edge of the segmentation in the right column of Fig. 4. This indicates that the resulting displacements inside the vessel wall are still more accurate.

In the ILT group, the mean motion drift was significantly decreased from 0.32 mm ± 0.18 mm to 0.07 mm ± 0.04 mm (p = 5·10^-4^). At the same time, the mean SNRe^circ^ (considering the total circumference of the aneurysm) was increased by 8.1 dB (p = 0.049), from -8.7 dB ± 8.3 dB using single-aperture US to -0.6 dB ± 6.9 dB using bistatic dual-aperture US. Using bistatic dual-aperture US imaging, the resulting strains display physiological patterns. In general, larger circumferential strains were measured near the lumen compared to the outer wall, which is expected with a more compliant ILT. This pattern is well visible in P33. In P29 and P30, a similar strain pattern was observed although the change in magnitude between the lumen and the outer border may be less pronounced at some locations. The corresponding cross-correlation values indicate that displacements at the lumen-thrombus border are harder to measure compared to locations in the ILT closer to the outer wall. Hence, there could be some inaccuracies in the measured strains at these locations. P33 is an example in which high cross-correlation values were measured within the ILT, displaying a high level of confidence for the measured strain values. Also in P20, the ILT was well visible on the US image and high cross-correlation values were found. However, this strain pattern was completely different since the largest circumferential strains were measured at the anterior part of the vessel wall, which also corresponds to the part of the aneurysm without thrombus.

Figure 5 shows the median strain pattern among all 40 patients, as measured in the aneurysm wall. The median circumferential strain was 0.019 at the anterior side and 0.005 at the posterior side. Two small peaks in circumferential strain were also measured at the lateral sides. The strain patterns were consistent among the 40 patients, especially at the anterior and posterior side, where minimal variation between patients was observed. No substantial differences were found when comparing median strain patterns between patients with and without ILT, and between patients with AAA diameter between 30 mm - 45 mm or >45 mm, however, small trends were observed, as shown in Fig. 5.

In patients with ILT, more homogeneous strain patterns were observed compared to patients without ILT, however, the difference in sample size could also be a factor. The variation in circumferential strain at the anterior and posterior sides was slightly larger among patients with small AAAs compared to large AAAs. In small AAAs, the IQR at the anterior side was (0.011, 0.026), compared to (0.017, 0.023) in large AAAs. On the posterior side, the IQR was equal to (0, 0.008) in small AAAs and (0.003, 0.007) in large AAAs. Furthermore, the median circumferential strain was slightly larger on the patient’s left side (4 o’clock) in smaller AAAs and AAAs without ILT, compared to AAAs with a diameter > 45 mm and AAAs with ILT.

## Discussion

In this study, dual-aperture US imaging was demonstrated in patients with an AAA, revealing large improvements in image quality and AAA visibility, enabling local motion and strain estimation at large imaging depths. Furthermore, dual-aperture US imaging also demonstrated the feasibility of performing strain imaging in the ILT. In the end, strain imaging inside the vessel wall and thrombus can provide new insights on the mechanical state of the AAA in an effort to improve rupture risk assessment.

**Fig. 6:**
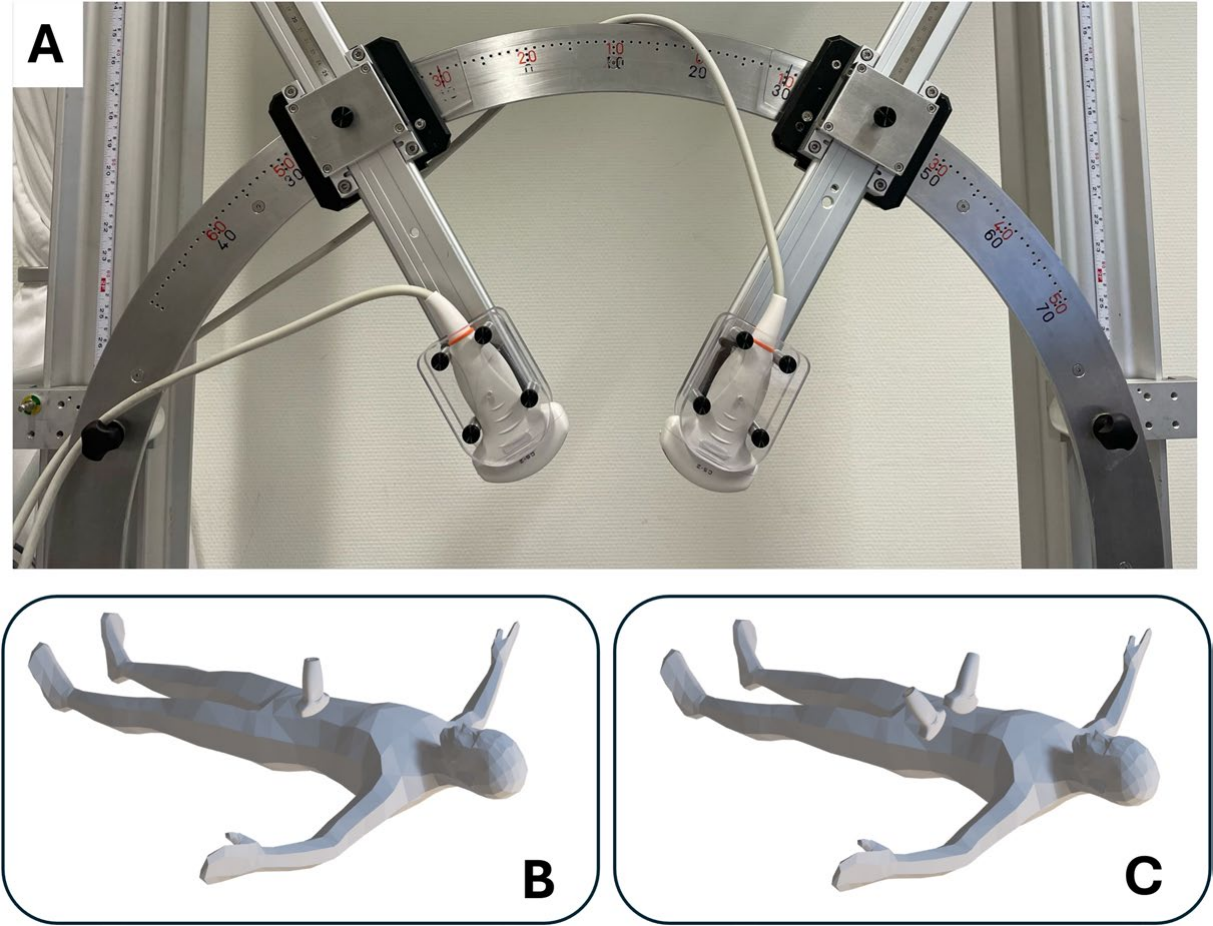
(**a**) Experimental arch used for the dual-aperture ultrasound measurements. (**b**) Illustration of the transversal single-probe ultrasound measurement of the AAA at 0$\phantom{0}^{\circ }$. (c) Illustration of the transversal bistatic dual-aperture ultrasound measurement. Illustration in (b) and (c) adapted from^45^.

**Fig. 7:**
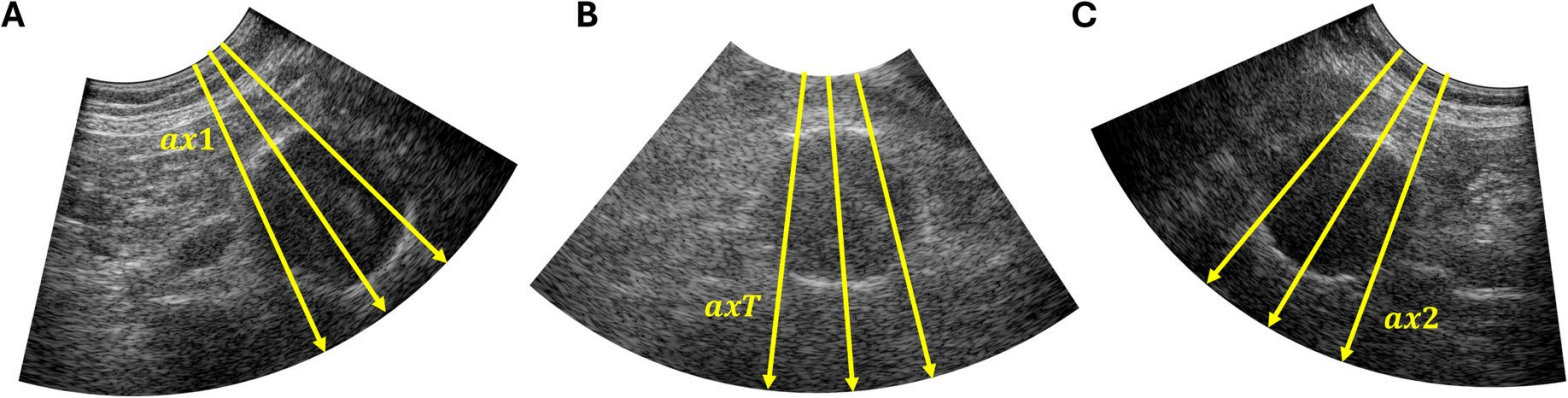
Sector grids used for reconstruction of the bistatic dual-aperture image data. (**a**) Sector grid used for reconstruction of the $T_1R_1$ data. (**b**) Sector grid used for reconstruction of the trans-probe data (i.e. $T_1R_2$ and $T_2R_1$ signals). (**c**) Sector grid used for reconstruction of the $T_2R_2$ data.

Both quantitative and qualitative analyses show that the image quality was significantly increased using bistatic dual-aperture US imaging, compared to single-aperture US. The analysis of the lumen-wall gCNR in the different regions across the circumference of the aorta showed that the lumen-wall contrast was significantly higher, compared to single-aperture US, especially at the lateral sides, indicating the benefit of performing dual-aperture US imaging on the visibility of the AAA geometry. In R1 and R5 the aneurysm wall was slightly better visible using single-aperture US, which can be explained by the fact that the single US probe is closer to these wall regions in the single-aperture acquisition, creating stronger reflections from the AAA wall. In the dual-aperture acquisition, the US probes are placed under an angle towards the side (Fig. 6), increasing the distance between the probes and the anterior and posterior segment. A third aperture could be considered, however, the addition of the trans-probe data in bistatic US imaging also results in additional reflections on the vessel-wall segments between the transducers (Fig. 7b), partially compensating the decrease in visibility.

To be able to fully visualize the entire circumference of the AAA, the two probes would ideally be placed under an angle of $90^{\circ }$. In practice, the optimal relative probe positioning is not only dependent on the angle between the probes but also on the image quality of the individual images. Choosing a smaller probe angle results in better visibility of the AAA on the US images of the individual probes. However, a larger angle results in a better angular coverage of the AAA wall by specular reflections if the images from both probes are combined. Currently, moving the probes towards the sides was often limited by the presence of the bowel or obstruction by the ribs in case the maximum AAA diameter was very proximal. The used inter-probe angles in our study ranged between 35$\phantom{0}^{\circ }$ and 80$\phantom{0}^{\circ }$. Independent of the used inter-probe angle, bistatic US imaging improved image quality compared to single-aperture US imaging. Eventually, the choices of the operator in the positioning of the probes have an influence on the final quality of the bistatic US image. Currently, positioning the two probes was not straightforward and required some experience. In clinical settings, dual-aperture ultrasound imaging would likely involve training of the sonographers to minimize the inter-operator variability in the probe positioning. In our study, all measurements were performed by one operator. However, investigating inter-operator variability would be important in the design of operator-friendly, multi-aperture imaging devices. In the future, it is also envisioned that these techniques could be integrated within the use of flexible transducer arrays^26^ using even more apertures, which would allow operator-independent, point-of-care monitoring of AAAs. Furthermore, the use of 3-D US imaging will also increase the field-of-view in the longitudinal direction of the AAA and remove the limitation of scanning in the same imaging plane^27,28^. If needed, deep learning techniques could further improve image quality or fill in the gaps in the future, as recently shown in US tomography^29^. In the end, the main source of variability in image quality in a patient cohort is determined by the anatomy of the patients and given the large range of inter-probe angles in our study, we assume that the inter-operator variability will be within the inter-patient range.

Bistatic dual-aperture US imaging significantly improved the visibility of the AAA wall and surrounding structures (abdominal muscles, spine). Improving the visibility of the ILT still remained to be challenging due to its low echogeneicity. Moreover, ILT visibility is hampered by clutter, especially in the upper part of the AAA. To further investigate the impact of the ILT on the mechanical state of the AAA wall, it is essential to improve the lumen-thrombus contrast on the US image, which would also benefit strain imaging. Since the presence of the ILT can be well identified using Color Doppler techniques, singular value decomposition (SVD) filters^30^ and clutter reduction techniques could potentially improve the ILT visibility or its delineation.

Comparing single-aperture US and bistatic US imaging, it is evident that bistatic US imaging yields more homogeneous strain patterns due to the mitigation of lateral motion estimation inaccuracies and drift, which resulted in improved tracking precision. The total motion drift in the vessel wall was decreased by 72% over all 40 patients, resulting in an increased SNRe^circ^ by 68%.

The resulting strain magnitudes are much smaller compared to healthy volunteers^25^, which is expected because of the increased stiffness of the AAA wall^16,18^. The strain patterns inside the AAA wall still showed some heterogeneity, in accordance with findings from previous literature^17,18,31^. A certain amount of heterogeneity in the aneurysm wall can be expected because of the large local variation in material properties and wall thickness, as well as the presence of calcifications^32^. The general strain pattern shown in Fig. 5 was consistent among the 40 AAA patients, as only small variations were present and no significant differences were found when analyzing the groups based on AAA diameter or the presence of ILT. For the presence of ILT, the results were limited by the sample size as there were only 7 patients without ILT. Regarding AAA diameter, these results are in line with previous findings from literature^18,31^, showing that the AAA diameter is not correlated with strain and stiffness. This suggests that biomechanical parameters such as the local differences in strain in each individual patient should be evaluated independent of AAA diameter when estimating AAA growth and rupture risk. Derwich et al.^31^ pointed out the importance of analyzing the heterogeneity of the strain pattern in combination with the mean circumferential strain, as it could provide additional information about the pathologic behavior of the aneurysm wall.

Lorenzen et al.^19^ is the only study to also report the mean local strain curves in AAA patients, as measured from 2D ultrasound. They reported a triphasic curve with two local maxima on each side of the spine and one at the right side of the anterior wall. Comparing these results to the results in our study, our median circumferential strain patterns were slightly more homogeneous, i.e. less strongly defined peaks. However, we also found two small peaks at the lateral sides of the wall and measured more strain at the anterior wall (1.9%), compared to posterior wall (0.5%). Moreover, we also observed a local maximum at the right side of the anterior wall (1 o’clock), especially in patients without thrombus. In this comparison, it is important to note that the principal strain direction may not always correspond to the circumferential direction. Regarding the differences in principal and circumferential strain in our study, the principal strains were slightly larger in magnitude compared to the reported circumferential strains. However, the patterns were similar.

In order to determine the exact clinical relevance of the measured local strain in the wall and ILT, it is important to perform longitudinal studies in the future that can gain better insights into the progression of local strain together with AAA growth and development in each individual patient. The anatomical and strain information from multi-aperture ultrasound images could then be used as an input for personalized biomechanical modeling, allowing an accurate estimation of other important biomechanical parameters such as the local material properties and wall stress^16^. This ultimately allows for patient-specific rupture risk assessment using multi-aperture ultrasound data.

Strain imaging in the wall was successful in all patients, and in 13 out of 25 patients with ILT, strain imaging was successfully performed in the entire AAA. In these 13 patients, the contrast on the bistatic US image data was sufficient to perform segmentation. There was one additional patient in which thrombus strain imaging was attempted but only the results inside the vessel wall were used, because of low cross-correlation values inside the thrombus. This study demonstrates the feasibility of assessing local strain information inside the ILT using dual-aperture US, which has not been attempted before using conventional US, because of the challenges involving the low echogenicity of the ILT. Therefore, this introduces new possibilities for mechanically characterizing the ILT and understanding its role in AAA development. Improving the ILT visibility, as mentioned above, is necessary to further improve these results and make thrombus strain imaging possible in larger patient cohorts.

In the ILT group, the mean motion drift was reduced by 78% over all patients, yielding increased SNRe^circ^ by 108% on average, compared to single-aperture US. Largest circumferential strains were measured inside the ILT near the lumen border, which decreased towards the outer wall, which was expected, but now also measured *in vivo*. After all, it is known from literature that the ILT is around 20 times more compliant compared to the AAA wall^33^. Moreover, the measured local changes in strain magnitude can also reflect local tissue composition as it is known that the luminal, medial, and abluminal layers of the ILT can have differences in strength and stiffness, depending on the type of aneurysm^6,34,35^. It is therefore interesting to further analyze the distribution of local strains in relation to local ILT thickness, and size and shape of the AAA on a larger dataset for future research.

In a few other patients, such as P15 and P20, largest circumferential strains were found in the thinnest part of the AAA wall instead of the lumen-thrombus border. According to Laplace theory, large circumferential strains can also be expected at the thinner part of the vessel wall in case of more homogeneous tissue properties, because the pressure gradient is largest at this location^36^. In P20, the ILT also appears to be more bright on the US image compared to other patients (Fig. 1), hence other tissue properties can be expected. In conclusion, thrombus strain imaging using dual-aperture US shows a lot of potential to measure local tissue mechanics, which could contribute to a better understanding on the morphology of the ILT.

It is evident that using multi-aperture ultrasound imaging, more homogeneous and precise strain estimates were obtained, across a larger part of the aneurysm’s circumference, compared to single-aperture ultrasound imaging. However, discussing the accuracy of the strain patterns remains difficult due to the lack of ground-truth data. Future research can further focus on validation of the strain patterns, for instance using realistic ultrasound simulations in which the ground truth displacements are known^22,37^. In the development of multi-aperture imaging devices, simulations also allow for the adjustment of the number of apertures to investigate the change in strain pattern in relation to the ground truth. It is expected that going from two to three or more apertures will have a smaller effect on the strain pattern compared to going from one to two apertures, since having only one aperture requires the use of the erroneous lateral displacement information. Using two apertures, the axial displacement information from multiple directions could be combined while mitigating the lateral displacements. Using three or more apertures, axial displacement information will be available in more directions, which has the potential to further improve the results. In addition to ultrasound simulations, validation of the strain patterns obtained using dual-aperture ultrasound imaging can also be performed using MRI^38^, FEM models and by comparison with CT data. For some patients, the CT data were available, which showed that regions of small to zero circumferential strain corresponded to calcified regions. However, since the US and the CT data could not be perfectly co-registered, these results were not included in the analysis and are subject to further investigation. Ultrasound speckle tracking and cine magnetic resonance imaging have not been compared side by side in patients with AAA, as MRI data for this group are scarce. However, such studies have been performed recently in the quantification of myocardial strain, showing moderate to good correlation between both methods^39^. One factor that could be of influence, especially for abdominal applications, is the probe pressure. In Bracco et al.^40^, it was found that probe pressure could result in an increase in the median strain and heterogeneity of the strain pattern and will therefore be important to take into account for future validations.

In clinical settings, dual-aperture imaging can be influenced by aberrations caused by the differences in the speed-of-sound of the medium. The impact of aberrations on dual-aperture US imaging has previously been investigated in^41^, where a model-based approach was implemented to correct for local speed-of-sound differences in dual-aperture imaging. In order to apply these methods *in vivo*, it is necessary to obtain the speed-of-sound distribution from the ultrasound images automatically, for instance using speed-of-sound imaging^42^. In this study, we have shown that even without the use of aberration correction, the B-mode image quality as well as the functional elastography measurements were significantly improved with bistatic imaging compared to single-aperture imaging. Although further improvements are expected with the integration of aberration correction, the effects of the different speed-of-sound in the media and possible registration errors were considered of minor influence.

Finally, it is important to note that the regions with a larger projection angle between the local radial direction and the axial direction, and lower image contrast, i.e., regions at 4 and 8 o’clock, can exhibit larger errors in the measured displacements and strain compared to the other regions in the vessel wall. In these regions, the accuracy of the speckle tracking will be influenced by the lack of reflections on the aortic wall and radial displacements can be overestimated during the angular decomposition of the measured axial displacements, as shown in^22^. Therefore, in all patients, a small amount of regularization was used to represent the strain pattern in the vessel wall and ILT. There was one patient in which the regularization filtered out the relatively fast movement of the ILT. Since the ILT is less stiff compared to the vessel wall, large displacements were measured as a result of the pressure pulse. Currently, the regularization strength was only a function of the initial geometry, and assumed a plane-strain Hookean material model^43^. However the ILT actually behaves according to a viscoelastic material model, depending on the type and layers of ILT material^44^, and its morphology can vary considerably between patients. In the future, the regularization could be adapted based on the location in the AAA, the presence of ILT, and the transducer configuration in each patient individually, since this determines if and how much regularization is needed.

To conclude, bistatic dual-aperture US imaging vastly improves image quality and strain estimation of AAAs compared to the use of conventional single-aperture US in a large part of the arterial wall, providing the clinician with new insights on the anatomical and mechanical state of AAAs. By compounding axial displacements from multiple directions, we show the feasibility of local strain quantification: not only in the vessel wall, but also inside the ILT, which introduces new possibilities for improving our understanding of the ILT and its role in aneurysm development, i.e., growth and weakening of the wall. Ultimately, the proposed multi-aperture technique is used to monitor patients to improve the assessment of rupture risk and the need for intervention.

## Methods

### Study design

The feasibility and performance of bistatic dual-aperture US imaging and elastography of the abdominal aorta was tested in patients with an AAA. We included 43 patients with varying age, body type, aneurysm geometry, and US image quality. Each patient had to meet all of the following criteria for inclusion in the study: AAA diameter $\ge$ 3.0 cm, the patient must be able to lie down on a bed during the examination, and patients must be able to hold their breath for five seconds.

The experiment protocol was approved by the local ethics review board of the Catharina Hospital Eindhoven on 22 December 2023 (ID-number: nWMO-2023.115) and written informed consent was obtained from each patient prior to scanning. This study was conducted in accordance with the principles of the Declaration of Helsinki and the EU GDPR (General Data Protection Regulation). The Medical research Ethics Committees United (MEC-U) confirmed that the Medical Research Involving Human Subjects Act (WMO) does not apply to this study. All acquisitions were performed at the Catharina Hospital Eindhoven in a three month period by a vascular sonographer with eight years of experience in AAA US scanning. Acquisitions were performed using the Verasonics Vantage 256 (Kirkland, Seattle, WA, USA), and two C5-2v curved array transducers with a center frequency of 3.7 MHz. A customized arch was designed on which the probe holders were attached (Fig. 6a). This mechanical arch allowed for free positioning of the probes under a certain angle, while ensuring that acquisitions were performed in the same imaging plane.

The patient was positioned in a supine position on an examination bed. Two acquisitions were performed on each patient:

- Transversal single-aperture ultrasound measurements at the location of the maximum anterior-posterior AAA diameter (Fig. 6b). An ultrafast acquisition scheme was used with 15 diverging waves between -12$\phantom{0}^{\circ }$ and 12$\phantom{0}^{\circ }$ at a frame rate of 130 Hz. The data were recorded for 5 seconds during breath-hold.
- Transversal bistatic dual-aperture ultrasound measurement at the location of the maximum diameter of the AAA using the optimal probe angles. The optimal probe angle depends on the anatomy and image quality of each patient (Fig. 6c). After adjusting for the optimal position of the transducers, based on the acquired images, data were recorded for approximately 5 seconds during breath-hold. Here, an interleaved acquisition scheme was used using 15 diverging waves between -12$\phantom{0}^{\circ }$ and 12$\phantom{0}^{\circ }$ per transducer. The resulting frame rate for each transducer and final compounded images was equal to 130 Hz^25^.

The total exam costs each patient an additional fifteen minutes of their time, directly after the regular US scan for AAA monitoring had been performed, thereby reducing the burden on the patients to a minimum.

The following demographic data were collected: age (years), gender (m/f), Body Mass Index (BMI, in kg/cm^2^), aortic diameter (mm) and the presence of ILT in the aneurysm, quantified by aortic lumen diameter (mm) and total ILT thickness (mm). The presence of the ILT was determined by Color Doppler US imaging (if performed), which was part of the regular AAA echographic exam, or from CT data of the same patient (if available).

### Image registration and fusion

In this study, the raw US channel data were collected. All post-processing steps were performed in MATLAB 2023a (The MathWorks, Natick, MA, United States). Registration and fusion of the images from the two US probes was performed in post-processing after the acquisition of the US data. Automatic probe localization was achieved by maximizing the signal coherence of the trans-probe data^25^.

After obtaining the relative probe positions, the individual four signals in bistatic US imaging were combined by means of coherent compounding on a grid with a spacing of $\frac{1}{4}$ of the wavelength ($\lambda$), i.e., 0.1 mm, using (1).

1 $$\begin{aligned} I_{bistatic} = I_{T_1R_1} + I_{T_2R_2} + \frac{1}{2}(I_{T_1R_2} + I_{T_2R_1}) \end{aligned}$$

Here, *T* indicates the transmitting transducer, which is either transducer 1 or 2, and *R* indicates the receiving transducer. The two trans-probe signals, which are the signals transmitted by one transducer and received by the other transducer, i.e., $T_1R_2$ and $T_2R_1$, contain similar information because of acoustic reciprocity^46^, therefore their relative contributions were averaged before summation with the single-probe signals ($T_1R_1$ and $T_2R_2$).

### Bistatic dual-aperture strain imaging

For the purpose of displacement estimation, the received RF data of the single-probe signals were reconstructed on a sector grid with respect to the receiving transducer (Fig 7a and c). The two trans-probe signals were reconstructed on a sector grid that was aligned with the virtual probe location, i.e., as if a third transducer was placed in between the two probes (see Fig 7b). Therefore, the axial direction of the sector grid was aligned with the location of the reflections on the aortic wall that occurred on the vessel wall segments between the transducers. The sector grid consisted of two lines per pitch in the lateral direction (pixel spacing: 0.41 - 0.54 mm/pixel, depending on the imaging depth), whereas the pixel spacing in the axial direction was set to $\frac{1}{8}\lambda$ (0.051 mm/pixel).

Manual segmentation of both the aortic wall and ILT was performed on the compounded bistatic dual-aperture US image. From the segmentation, a mesh was created with 17 by 151 points in the radial and circumferential directions, respectively. In the absence of ILT, or in case the ILT was not visible on the US images, the vessel wall segmentation was created by delineating only the lumen-wall border after which the outer border was defined by extruding the luminal contour radially with a uniform wall thickness of 2 mm. Here, the resulting mesh size was 5 by 101 points.

Displacement estimation was performed on the individual four signals using a 2-D coarse-to-fine speckle-tracking algorithm^47^. The block matching was performed on the IQ-data using complex normalized cross-correlation. As a first step, the coarse displacements were estimated using a kernel size of 2.6 mm $\times$ 4.5 - 5.9 mm (axial $\times$ lateral). Secondly, the displacements found in the first step were refined using a kernel size of 0.8 mm $\times$ 2.1 - 2.7 mm. The search area was defined to be 3.0 mm $\times$ 4.9 - 6.5 mm for the coarse displacements and 1.0 mm $\times$ 2.5 - 3.2 mm for the fine displacements. As a last step, the axial and lateral displacements were filtered with a median filter of 0.6 mm $\times$ 4.5 - 5.9 mm (11 $\times$ 11 pixels).

**Fig. 8:**
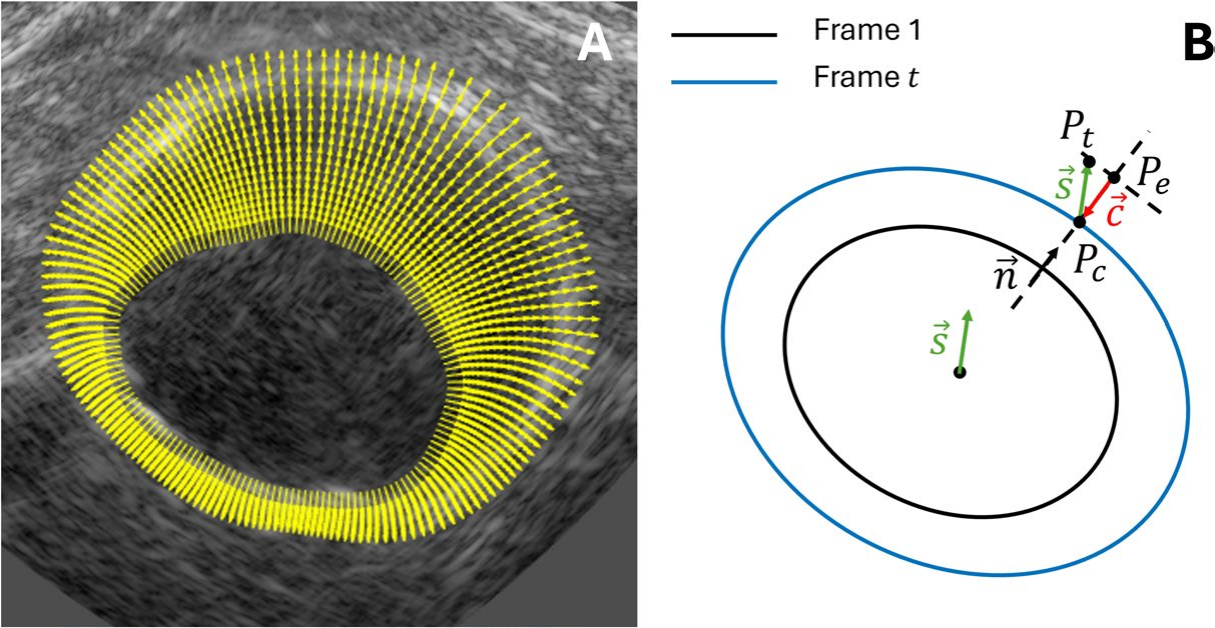
(**a**) Local radial directions of an AAA segmentation including the vessel wall and thrombus. (**b**) Illustration of the correction for the midpoint shift, that occurred between two frames, in the calculation of the strains. $P_c$ represents a point in the corrected contour of the AAA wall, i.e., compensated for the rigid body motion. This point originates from $P_t$ by midpoint shift $\vec {s}$. Because of radial projection, this point is estimated to be at $P_e$. $\vec {c}$ is the correction that is applied, in the normal directions $\vec {n}$ of the AAA wall at end-diastole.

To fully leverage the multi-aperture image data, the axial displacements were estimated in each of the four datasets and compounded. The lateral displacement estimation results were not taken into account, because of the well-known drawbacks, i.e., lack of phase information and low resolution of the RF signals in the lateral direction. The axial displacement fields $u_{ax}$ were radially projected according to $u_{rad} = u_{ax}/\cos (\theta )$^48^, in which $\theta$ represents the angle between the axial direction and the local radial direction of the aorta. To accurately fit the local geometry of the aneurysm, the local coordinate system was derived from the local normal directions of the segmentation mesh, as shown in Fig. 8a.

Angular weighted normalized masks *M* were defined to retrieve the most accurate displacement estimates from each US signal^22,24^. Again, the radial displacements fields from $T_1R_2$ and $T_2R_1$ were averaged before summation with the radial displacement fields from $T_1R_1$ and $T_2R_2$, because these signals contain similar image information. Finally, the radial displacements were compounded according to the following fusion rule:

2 $$\begin{aligned} u_{rad} = M_{T_1R_1} (u_{rad,T_1R_1}) + M_{T_2R_2}(u_{rad,T_2R_2}) + \frac{1}{2} M_{T_1R_2} (u_{rad,T_1R_2}) + \frac{1}{2} M_{T_2R_1} (u_{rad,T_2R_1}) \end{aligned}$$

The resulting compounded displacement field was applied to the segmentation of the vessel wall (and ILT if applicable) to track the wall motion over all frames.

The displacements of the tracked coordinates were calculated with respect to the first frame, after which a 2D least-squares strain estimator, described in Lopata et al.^49^, was applied. With a mesh size of 5 radial $\times$ 101 circumferential points, a strain kernel of 5 radial $\times$ 9 circumferential mesh points was applied. In patients in which not only the vessel wall, but also the ILT was tracked, an adaptive strain kernel was applied because of the large difference in spacing between the inner and outer borders of the segmentation. A strain kernel of 9 radial $\times$ 15 circumferential mesh points was applied at the outer border. Toward the inner border, the number of mesh points in the circumferential direction was increased, making sure that the size of the strain kernel in millimeters, depending on the spacing between the mesh points in the segmentation, remained approximately constant.

Before calculating the local strains, a correction is performed on the tracked *x*- and *z*-coordinates of the vessel wall segmentation. This allows to take the displacement of the midpoint into account that occurred between each reconstructed frame. With radial expansion of the aorta, the centerpoint of the aorta shifts slightly towards the anterior direction, because the expansion to the posterior side is limited by the presence of the spine^50,51^. Therefore, the local radial and circumferential directions of the AAA change during the cardiac cycle and local radial displacements could be over- or underestimated during radial projection^48^. The midpoints were calculated for each frame by taking the average of the *x*- and *z*-coordinates of the segmentation (center of mass). A smoothing spline was fitted through the tracked midpoints to mitigate small variations. The midpoint correction is illustrated in Fig. 8b. The tracked coordinates of the vessel wall were compensated by $\vec {c}$, which is the shift of the midpoint $\vec {s}$, that occurred between two frames, projected onto the local radial directions of the AAA wall $\vec {n}$, as shown in Fig. 8:

3 $$\begin{aligned} \vec {c} = -(\vec {s} \cdot \vec {n})\vec {n} \end{aligned}$$

As a last step, the roughness of the resulting displacement field was regularized using a method that constrains the thin plate bending energy^43^. As a criterion, the maximum allowed scaled root mean squared roughness of the frame-to-frame displacements is used:

4 $$\begin{aligned} E^*_{bend} \le \xi ^* \cdot \gamma \cdot RMS(u_r). \end{aligned}$$

Here, $RMS(u_r)$ denotes the root mean square radial displacements, $E^*_{bend}$ represents the average bending energy, $\gamma$ is a variable that is only a function of the initial geometry^43^, and $\xi ^*$ is the parameter to control the regularization strength. Only a value of $\xi ^* <$ 1 leads to an active constraint on the $E^*_{bend}$. In this study, slight regularization was applied in both single-aperture US and bistatic dual-aperture US configurations by choosing a value for $\xi ^*$ of 0.9. In this way, the tracked segmentation is regularized (i.e., has an effect on the motion drift), while local variation in strain are preserved.

### Data analysis

#### Image quality

The generalized contrast-to-noise ratio (gCNR)^52^ was computed as a robust measure for the contrast between the lumen and the vessel wall on the envelope-detected US images. In this analysis, any potential ILT was included in the lumen segmentation because of the poor visibility of the ILT in many of the US images. The aforementioned vessel wall segmentation and lumen segmentation were converted into binary masks for the calculation of the gCNR. The binary mask of the lumen was eroded by a disk-shaped structuring element with a radius of 0.6 mm to ensure that the specular reflections from the vessel wall were not included. For this analysis, the segmentation of the lumen-wall border was repeated three times and the resulting gCNR values were averaged to obtain reliable results.

The region of interest (ROI) of the vessel wall was divided in 8 sections of 45$\phantom{0}^{\circ }$, to allow for a regional analysis of the gCNR across the vessel’s circumference. Region 1 and region 5 were defined to align with the wave propagation direction for the single probe configuration (0$\phantom{0}^{\circ }$ angle equal to the principal axis of the transducer). The other regions were defined in a clockwise manner. Comparisons in the gCNR for each region between single-aperture US and bistatic dual-aperture US imaging were made with the Wilcoxon signed rank test, due to non-normal distributions.

Apart from the image quality assessment using the gCNR, a visual assessment was performed to examine the differences between single-aperture US and bistatic dual-aperture US scans. For the visual assessment, five professionals (three vascular technologists, one vascular surgeon and one researcher) independently scored the US images on an image quality Likert scale, based on their perception of the image quality. All images were displayed randomly, ensuring that the reviewers were blinded to the type of US scanning method (single-aperture or dual-aperture US). The scale of the visual assessment was divided into five points:

1. Very poor image quality of the aortic wall. Not confident about aortic wall location.
2. Poor image quality of the aortic wall. Reasonable reflections to find a part of the aortic wall.
3. Fair image quality of the aortic wall. Visible wall reflections, moderate lumen-wall contrast
4. Good image quality of the aortic wall. Clear wall reflections, good lumen-wall contrast
5. Excellent image quality of the aortic wall. Excellent reflections to find the entire aortic wall.

The visual assessment results were expressed as the median score with interquartile range (IQR). Comparison of visual image quality between single-aperture US and bistatic US was made with the Wilcoxon signed rank test. In this analysis, differences were considered significant for p<0.05.

#### Tracking performance

For the analysis of the tracking performance, the mean motion drift (MD) was used. The tracking precision was calculated as the distance between the *n* points in the middle wall layer of the vessel segmentation at the start and the end of a complete cardiac cycle, at end-diastole. With a higher precision of the estimated displacements, the MD decreases, since the aortic wall comes back to the same position after tracking one cardiac cycle. The MD is defined in (5), where $x_i$ and $z_i$ denote the positions in the aortic wall at the starting frame, i.e. first end-diastole (ed1) and ending frame, i.e. second end-diastole (ed2). These two frames were selected from the M-mode of the acquired US frames.

5 $$\begin{aligned} \text {MD} = \frac{1}{n} \sum ^n_{i=1} \sqrt{(x_{i,ed1}-x_{i,ed2})^2 + (z_{i,ed1}-z_{i,ed2})^2} \end{aligned}$$

The strain estimation precision was quantified by the elastographic signal-to-noise ratio (SNRe), defined by (6), using the mean ($\mu _{\varepsilon }$) and the standard deviation ($\sigma _{\varepsilon }$) of the circumferential strain estimates as measured in the middle layer of the segmented wall.

6 $$\begin{aligned} SNR_e = 20\log _{10}\frac{\mu _\varepsilon }{\sigma _\varepsilon } \end{aligned}$$

To compare the means of these metrics between single-aperture US and bistatic dual-aperture US imaging across the total circumference of the vessel wall, Paired Samples t-tests were performed. If the differences were not normally distributed, a paired Wilcoxon signed rank test was used. Differences were considered significant for p<0.05.

## Supplementary Information

Supplementary Information 1.

Supplementary Information 2.

Supplementary Material 3

## Data Availability

The data that support the findings of this study are available from the Eindhoven University of Technology and Catharina Hospital Eindhoven but restrictions apply to the availability of these data, which were used under license for the current study, and so are not publicly available. Data are however available from the authors upon reasonable request and with permission of the local ethics review board of the Catharina Hospital Eindhoven.

## References

[1] Ullery, B. W., Hallett, R. L. & Fleischmann, D. Epidemiology and contemporary management of abdominal aortic aneurysms. *Abdom. Radiol.* **43**, 1032–1043 (2018). 10.1007/s00261-017-1450-7 29313113

[2] Reimerink, J. J., van der Laan, M. J., Koelemay, M. J., Balm, R. & Legemate, D. A. Systematic review and meta-analysis of population-based mortality from ruptured abdominal aortic aneurysm. *J. British Surg.* **100**(11), 1405–1413 (2013). 10.1002/bjs.9235 24037558

[3] Wanhainen, A. et al. Editor’s Choice - European Society for Vascular Surgery (ESVS) 2024 Clinical Practice Guidelines on the Management of Abdominal Aorto-Iliac Artery Aneurysms. *Eur. J. Vasc. Endovasc. Surg.* **67**, 192–331 (2024). 38307694 10.1016/j.ejvs.2023.11.002

[4] Lederle, F. A. et al. Immediate repair compared with surveillance of small abdominal aortic aneurysms. *NEJM* **346**, 1437–1444 (2002). 12000813 10.1056/NEJMoa012573

[5] Lederle, F. A. et al. Rupture rate of large abdominal aortic aneurysms in patients refusing or unfit for elective repair. *JAMA* **287**, 2968–2972 (2002). 12052126 10.1001/jama.287.22.2968

[6] Tong, J. & Holzapfel, G. A. Structure, mechanics, and histology of intraluminal thrombi in abdominal aortic aneurysms. *Ann. Biomed. Eng.* **43**, 1488–1501 (2015). 25986953 10.1007/s10439-015-1332-5

[7] Xenos, M. & Bluestein, D. *In Biomechanics and Mechanobiology of Aneurysms* (Springer, Berlin Heidelberg, 2011).

[8] Zambrano, B. A. et al. Association of intraluminal thrombus, hemodynamic forces, and abdominal aortic aneurysm expansion using longitudinal CT images. *Ann. Biomed. Eng.* **44**, 1502–1514 (2016). 26429788 10.1007/s10439-015-1461-x PMC4826625

[9] Speelman, L. et al. The mechanical role of thrombus on the growth rate of an abdominal aortic aneurysm. *J Vasc. Surg.* **51**, 19–26 (2010). 19944551 10.1016/j.jvs.2009.08.075

[10] Vorp, D. A. et al. Association of intraluminal thrombus in abdominal aortic aneurysm with local hypoxia and wall weakening. *J Vasc. Surg.* **34**, 291–299 (2001). 11496282 10.1067/mva.2001.114813

[11] Kok, A. M. et al. Feasibility of wall stress analysis of abdominal aortic aneurysms using three-dimensional ultrasound. *J Vasc. Surg.* **61**, 1175–1184 (2015). 25701496 10.1016/j.jvs.2014.12.043

[12] Van Disseldorp, E. M. J. et al. Patient specific wall stress analysis and mechanical characterization of abdominal aortic aneurysms using 4d ultrasound. *Eur. J. Vasc. Endovasc. Surg.* **52**, 635–642 (2016). 27665991 10.1016/j.ejvs.2016.07.088

[13] Wittek, A. et al. In vivo determination of elastic properties of the human aorta based on 4D ultrasound data. *J. Mech. Behav. Biomed. Mater.* **27**, 167–183 (2013). 23668998 10.1016/j.jmbbm.2013.03.014

[14] Karatolios, K. et al. Method for aortic wall strain measurement with three-dimensional ultrasound speckle tracking and fitted finite element analysis. *Ann. Thorac. Surg* **96**, 1664–1671 (2013). 23998405 10.1016/j.athoracsur.2013.06.037

[15] Derwich, W. et al. High resolution strain analysis comparing aorta and abdominal aortic aneurysm with real time three dimensional speckle tracking ultrasound. *Eur. J. Vasc. Endovasc. Surg.* **51**, 187–193 (2016). 26391962 10.1016/j.ejvs.2015.07.042

[16] Van Disseldorp, E. M. J., Petterson, N. J., van de Vosse, F. N., van Sambeek, M. R. H. M. & Lopata, R. G. P. Quantification of aortic stiffness and wall stress in healthy volunteers and abdominal aortic aneurysm patients using time-resolved 3D ultrasound: a comparison study. *Eur. Heart J. Cardiovasc. Imag.* **20**, 185–191 (2019). 10.1093/ehjci/jey051 29618036

[17] Bracco, M. I. et al. Fast strain mapping in abdominal aortic aneurysm wall reveals heterogeneous patterns. *Front. Physiol.* **14**, 1163204 (2023). 37362444 10.3389/fphys.2023.1163204 PMC10285457

[18] Zottola, Z. R. et al. Intermediate pressure-normalized principal wall strain values are associated with increased abdominal aortic aneurysmal growth rates. *Front. Cardiovasc. Med.* **10**, 1232844 (2023). 37719977 10.3389/fcvm.2023.1232844 PMC10501562

[19] Lorenzen, U. S. et al. Strain patterns with ultrasound for assessment of abdominal aortic aneurysm vessel wall biomechanics. *Ultrasound Med. Biol.* **51**, 112–119 (2025). 39366791 10.1016/j.ultrasmedbio.2024.09.014

[20] Matthews, E. O. et al. The reproducibility of measuring maximum abdominal aortic aneurysm diameter from ultrasound images. *Ultrasound J.* **13**, 1–6 (2021). 33646456 10.1186/s13089-021-00211-z PMC7921236

[21] Petterson, N. J., van Sambeek, M. R. H. M., van de Vosse, F. N. & Lopata, R. G. P. Enhancing lateral contrast using multi-perspective ultrasound imaging of abdominal aortas. *Ultrasound Med. Biol.* **47**, 535–545 (2021). 33349515 10.1016/j.ultrasmedbio.2020.09.023

[22] Van Hal, V. H. J. et al. Multiperspective bistatic ultrasound imaging and elastography of the ex vivo abdominal aorta. *IEEE Trans. Ultrason. Ferroelectr. Freq. Control* **69**, 604–616 (2021). 10.1109/TUFFC.2021.3128227 34780324

[23] Burkholder, R., Gupta, L. & Johnson, J. Comparison of monostatic and bistatic radar images. *IEEE Antennas. Propag. Mag.* **45**, 41–50 (2003).

[24] De Hoop, H. et al. Multiperspective ultrasound strain imaging of the abdominal aorta. *IEEE Trans. Med. Imag.* **39**, 3714–3724 (2020). 10.1109/TMI.2020.3003430 32746118

[25] Van Hal, V. H. J., de Hoop, H., van Sambeek, M. R. H. M., Schwab, H.-M. & Lopata, R. G. P. In vivo bistatic dual-aperture ultrasound imaging and elastography of the abdominal aorta. *Front. Physiol.* **15**, 1320456 (2024). 38606009 10.3389/fphys.2024.1320456 PMC11007781

[26] Van Neer, P. L. et al. Flexible large-area ultrasound arrays for medical applications made using embossed polymer structures. *Nature Commun.* **15**, 280 (2024). 38555281 10.1038/s41467-024-47074-1 PMC10981753

[27] De Hoop, H., Vermeulen, M., Schwab, H.-M. & Lopata, R. G. Coherent bistatic 3-D ultrasound imaging using two sparse matrix arrays. *IEEE Trans. Ultrason. Ferroelectr. Freq. Control* **70**, 182–196 (2022). 10.1109/TUFFC.2022.3233158 37027570

[28] Jansen, L. C., Fekkes, S., Schwab, H.-M. & Lopata, R. G. Increasing abdominal aortic aneurysm curvature visibility using 3D dual probe bistatic ultrasound imaging combined with probe translation. *Ultrasonics* **139**, 107284 (2024). 38458061 10.1016/j.ultras.2024.107284

[29] Liu, Z. et al. *IEEE Int. Ultrason. Symp.* **2021**, 1–4 (2021).

[30] Van Hees, R. et al. SVD-based filtering to detect intraplaque hemorrhage using single wavelength photoacoustic imaging. *J. Biomed. Opt* **26**, 116003–116003 (2021). 34743446 10.1117/1.JBO.26.11.116003 PMC8571807

[31] Derwich, W. et al. Changes in aortic diameter and wall strain in progressing abdominal aortic aneurysms. *J Ultrasound Med.* **42**, 1737–1746 (2023). 36794590 10.1002/jum.16193

[32] Barrett, H. E. et al. On the influence of wall calcification and intraluminal thrombus on prediction of abdominal aortic aneurysm rupture. *J Vasc. Surg.* **67**, 1234–1246 (2018). 28899569 10.1016/j.jvs.2017.05.086

[33] Wittek, A. et al. Image, geometry and finite element mesh datasets for analysis of relationship between abdominal aortic aneurysm symptoms and stress in walls of abdominal aortic aneurysm. *Data in Brief* **30**, 105451 (2020). 32322616 10.1016/j.dib.2020.105451 PMC7171530

[34] Gasser, T. C., Görgülü, G., Folkesson, M. & Swedenborg, J. Failure properties of intraluminal thrombus in abdominal aortic aneurysm under static and pulsating mechanical loads. *J Vasc. Surg.* **48**, 179–188 (2008). 18486417 10.1016/j.jvs.2008.01.036

[35] O’Leary, S. A., Kavanagh, E. G., Grace, P. A., McGloughlin, T. M. & Doyle, B. J. The biaxial mechanical behaviour of abdominal aortic aneurysm intraluminal thrombus: Classification of morphology and the determination of layer and region specific properties. *J. Biomech.* **47**, 1430–1437 (2014). 24565182 10.1016/j.jbiomech.2014.01.041

[36] McGloughlin, T. *Biomechanics and mechanobiology of aneurysms* (Springer, Berlin Heidelberg, 2011).

[37] Van Aarle, D.; A.; C. et al. Numerical simulation of intravascular ultrasound images based on patient-specific computed tomography. IEEE Transactions on Ultrasonics, Ferroelectrics, and Frequency Control, 1–1 (2024). 10.1109/TUFFC.2024.3523037 40030621

[38] Satriano, A., Rivolo, S., Martufi, G., Finol, E. A. & Di Martino, E. S. In vivo strain assessment of the abdominal aortic aneurysm. *J Biomech.* **48**, 354–360 (2015). 25497379 10.1016/j.jbiomech.2014.11.016

[39] Brandt, Y. et al. Quantification of left ventricular myocardial strain: Comparison between MRI tagging, MRI feature tracking, and ultrasound speckle tracking. *NMR in Biomed.* **37**(9), e5164 (2024). 10.1002/nbm.5164 38664924

[40] Bracco, M. I., Yousefi, A. A. K., Rouet, L. & Avril, S. Ultrasound probe pressure affects aortic wall stiffness: a patient-specific computational study in abdominal aortic aneurysms. *Annals Biomed. Eng.* **53**(1), 1–12 (2024). 10.1007/s10439-024-03608-8 PMC11782392 39230788

[41] Van Hal, V. H. J., Muller, J.-W., Van Sambeek, M. R. H. M., Lopata, R. G. P. & Schwab, H.-M. An aberration correction approach for single and dual aperture ultrasound imaging of the abdomen. *Ultrasonics* **131**, 106936 (2023). 36774785 10.1016/j.ultras.2023.106936

[42] Jaeger, M. et al. Pulse-echo speed-of-sound imaging using convex probes. *Phys. Med. Biol.* **67**, 215016 (2022). 10.1088/1361-6560/ac96c6 36179699

[43] Muller, J.; W., Wu, M., Rutten, M.; C.; M., Van Sambeek, M.; R.; H.; M. & Lopata, R.; G.; P. Lagrangian framework to regularise ultrasound strain data of the large arteries using a finite element method. Under review (2025).

[44] Johnson, S. et al. Review of mechanical testing and modelling of thrombus material for vascular implant and device design. *Ann. Biomed. Eng.* **45**, 2494–2508 (2017). 28849421 10.1007/s10439-017-1906-5

[45] Lasso, A. et al. PLUS: Open-source toolkit for ultrasound-guided intervention systems. *IEEE Trans. Biomed. Eng.* **6**(10), 2527–2537 (2014). 10.1109/TBME.2014.2322864 PMC4437531 24833412

[46] Devaney, A. J. *Mathematical foundations of imaging, tomography and wavefield inversion* (Cambridge University Press, Cambridge, 2012).

[47] Lopata, R. G. P. et al. Performance evaluation of methods for two-dimensional displacement and strain estimation using ultrasound radio frequency data. *Ultrasound Med. Biol.* **35**, 796–812 (2009). 19282094 10.1016/j.ultrasmedbio.2008.11.002

[48] Hansen, H. H. G., Lopata, R. G. P., Idzenga, T. & de Korte, C. L. An angular compounding technique using displacement projection for noninvasive ultrasound strain imaging of vessel cross-sections. *Ultrasound Med. Biol.* **36**, 1947–1956 (2010). 20850217 10.1016/j.ultrasmedbio.2010.06.008

[49] Lopata, R. G. P., Hansen, H. H. G., Nillesen, M. M., Thijssen, J. M. & De Korte, C. L. Comparison of one-dimensional and two-dimensional least-squares strain estimators for phased array displacement data. *Ultrason. Imaging* **31**, 1–16 (2009). 19507679 10.1177/016173460903100101

[50] Nagy, R., Csobay-Novák, C., Lovas, A., Sótonyi, P. & Bojtár, I. Non-invasive in vivo time-dependent strain measurement method in human abdominal aortic aneurysms: Towards a novel approach to rupture risk estimation. *J. Biomech.* **48**, 1876–1886 (2015). 25980555 10.1016/j.jbiomech.2015.04.030

[51] Iffrig, E., Wilson, J. S., Zhong, X. & Oshinski, J. N. Demonstration of circumferential heterogeneity in displacement and strain in the abdominal aortic wall by spiral cine DENSE MRI. *J. Magn. Resonan. Imag.* **49**, 731–743 (2019). 10.1002/jmri.26304 30295345

[52] Rodriguez-Molares, A. et al. The generalized contrast-to-noise ratio: A formal definition for lesion detectability. *IEEE Trans. Ultrasonics, Ferroelect., Frequency Control* **67**, 745–759 (2019). 10.1109/TUFFC.2019.2956855 PMC8354776 31796398

