# Supplementary figures and images for: Strain imaging in abdominal aortic aneurysms using bistatic dual-aperture ultrasound
Source: Sci Rep. 2025 Nov 13;15:39892. doi: 10.1038/s41598-025-23710-8 (PMC12615740; doi:10.1038/s41598-025-23710-8)

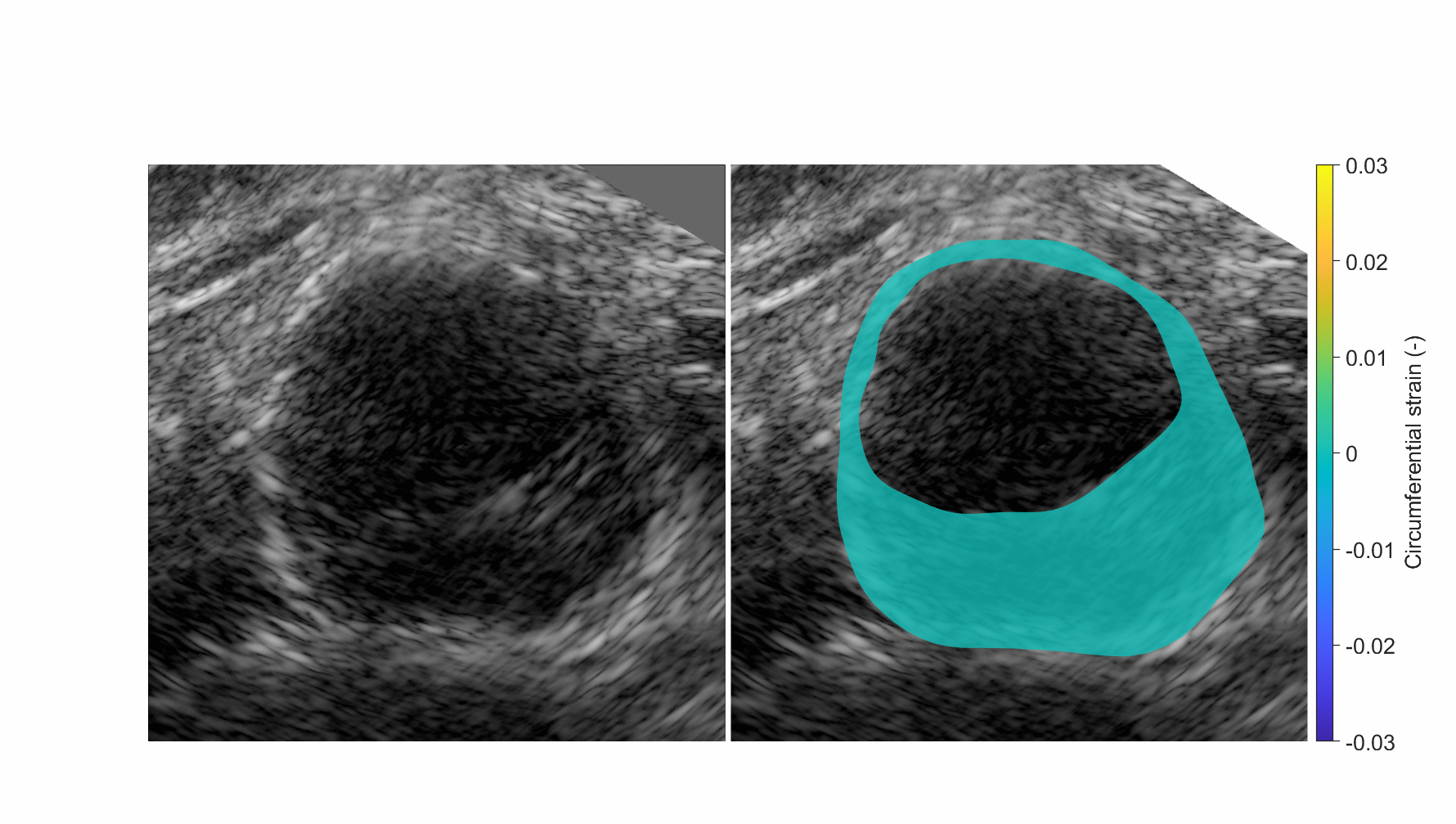

Supplement: Supplementary file 1 — Supplementary Information 1. [file 41598_2025_23710_MOESM1_ESM.gif]

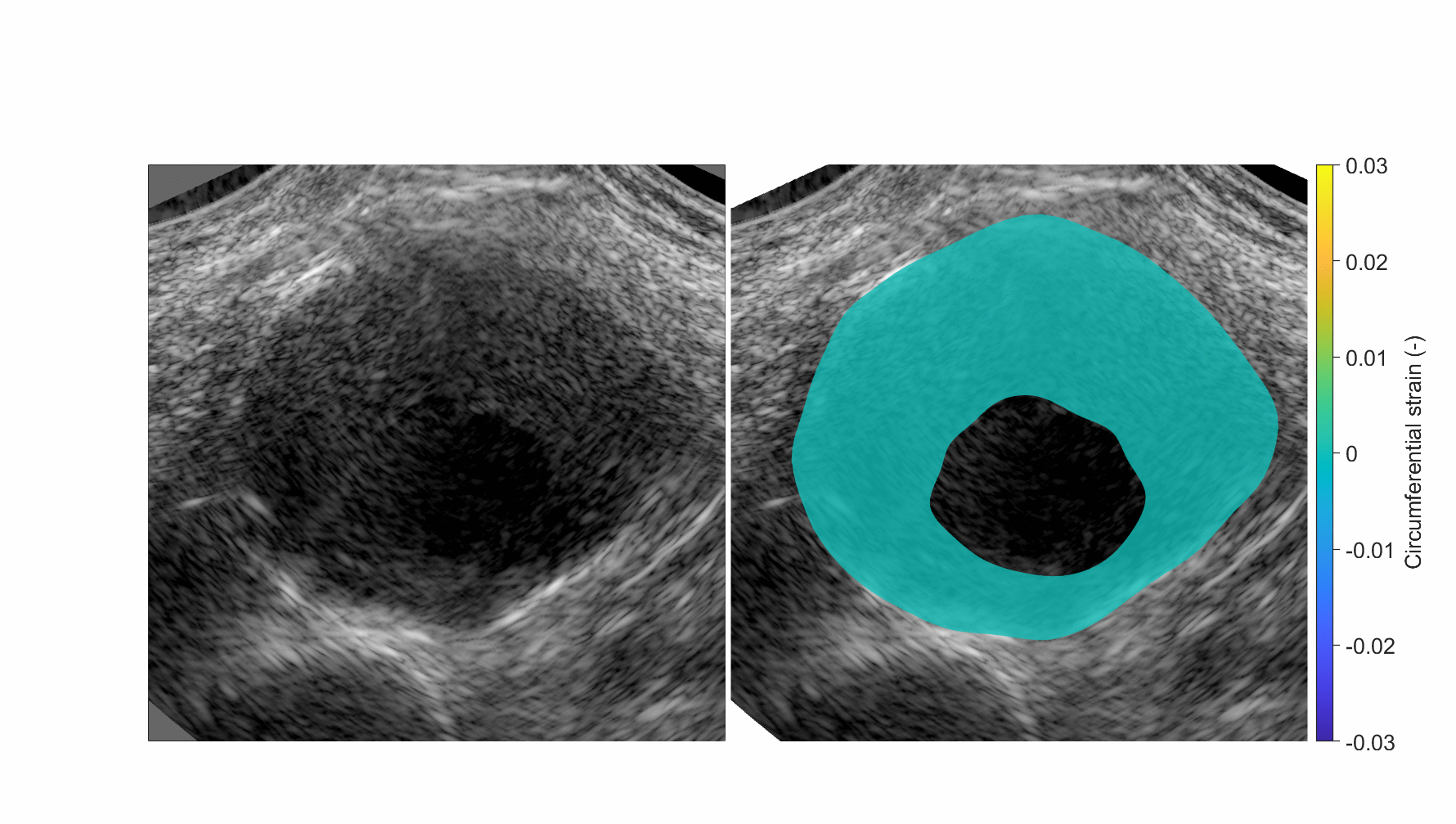

Supplement: Supplementary file 2 — Supplementary Information 2. [file 41598_2025_23710_MOESM2_ESM.gif]
